# Case Report: The First Report of *NUP214-ABL1* Fusion Gene in Acute Myeloid Leukemia Patient Detected by Next-Generation Sequencing

**DOI:** 10.3389/fonc.2021.706798

**Published:** 2021-07-08

**Authors:** Huan-Ping Wang, Jun-Jun He, Qiao-Yun Zhu, Lin Wang, Jian-Hu Li, Jian-Song Huang, Wan-Zhuo Xie, Hong-Hu Zhu, Jie Jin

**Affiliations:** ^1^ Department of Hematology, The First Affiliated Hospital, College of Medicine, Zhejiang University, Hangzhou, China; ^2^ Zhejiang Province Key Laboratory of Hematology Oncology Diagnosis and Treatment, Hangzhou, China; ^3^ Zhejiang Provincial Key Laboratory of Pancreatic Disease, The First Affiliated Hospital, College of Medicine, Zhejiang University, Hangzhou, China; ^4^ Central Laboratory, The First Affiliated Hospital, College of Medicine, Zhejiang University, Hangzhou, China

**Keywords:** *ABL1*, *NUP214-ABL1*, acute myeloid leukemia, tyrosine kinase, next-generation sequencing

## Abstract

The *NUP214-ABL1* fusion gene is a constitutively active tyrosine kinase that can be detected in 6% of T-cell acute lymphoblastic leukemia (T-ALL) patients, and it can also be found in B-cell acute lymphoblastic leukaemia (B-ALL). However the *NUP214-ABL1* fusion in acute myeloid leukemia (AML) has not yet been reported. Up to now, the sensitivity of *NUP214-ABL1*-positive patients to tyrosine kinase inhibitor (TKI) is still controversial. Here we report the first case of an AML patient carrying *NUP214-ABL1* fusion gene. The conventional AML chemotherapy regimen for the patient was successful. Identification of additional AML patients with *NUP214-ABL1* fusion gene will provide treatment experience and prognostic evaluation.

## Introduction

Identifying genetically targetable abnormalities that may respond to targeted therapy is clinically important in the context of precision medicine. Among all the fusion genes involving *ABL1* rearrangement, the *NUP214-ABL1* is the second common fusion in haematologic malignancies. About 6% of T-cell acute lymphoblastic leukaemia (T-ALL) patients carry this fusion gene (1). It can also be found in B-cell acute lymphoblastic leukaemia (B-ALL) (2, 3). However, as far as we know, the *NUP214-ABL1* fusion gene has never been reported in acute myeloid leukaemia (AML).

*NUP214-ABL1*, a constitutively active tyrosine kinase, is a potential target of tyrosine kinase inhibitors (TKIs) (4, 5). However, the therapeutic effect of TKI for the *NUP214-ABL1*-positive patients is controversial due to limited clinical experience (6–11). Some studies have shown that TKI monotherapy or combined chemotherapy is effective for *NUP214-ABL1*-positive patients (6, 10–13). However, other studies have shown that *NUP214-ABL1*-positive patients had no response to TKI therapy or developed resistance to TKIs post relapse (3, 7–9).

Given that many *NUP214-ABL1*-positive patients were diagnosed retrospectively and had already received classical treatment (14), more *NUP214-ABL1* cases are needed to guide clinicians caring for this subgroup of patients. Here, we describe the first case of an AML patient with the *NUP214-ABL1* fusion gene detected by next-generation sequencing (NGS).

## Case Presentation

**Figure 1:**
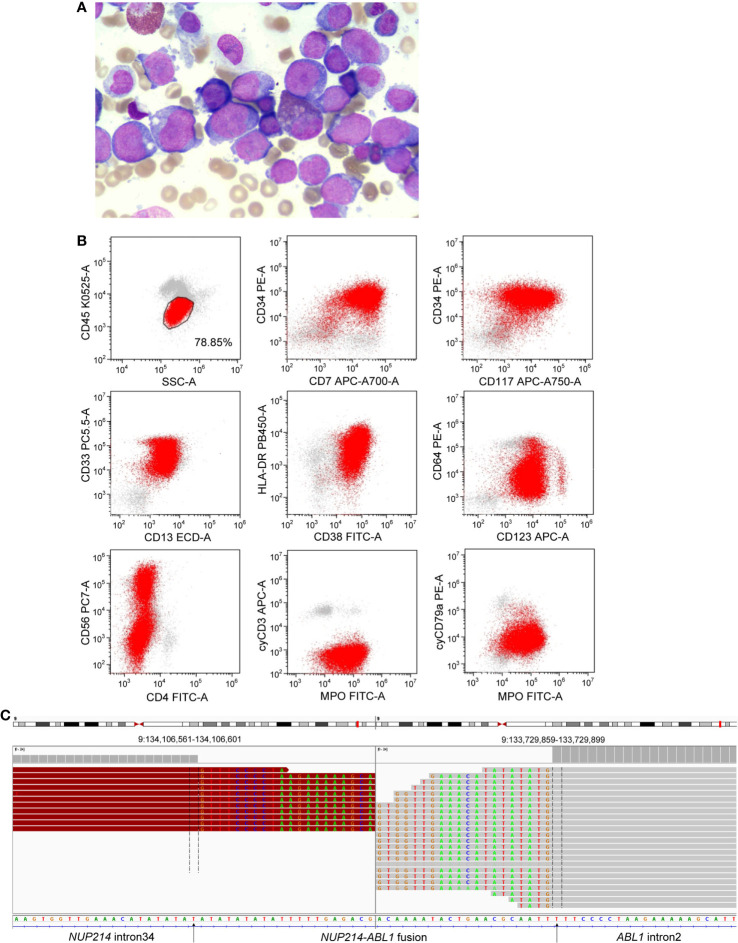
**(A)** Morphological observation showed blasts in a bone marrow smear. **(B)** Immunophenotype of AML was determined by flow cytometry. **(C)** Next-generation sequencing revealed a breakpoint in intron 34 of *NUP214* and *a* breakpoint in intron 2 of *ABL1*, resulting in the *NUP214*-*ABL1* fusion gene.

In January 2021, a 42-year-old male patient was admitted to our hospital due to skin bleeding. The patient’s blood count was as follows: white blood cells (WBCs) 82.5×10^9^/L (differential: neutrophils 2.7%, eosinophils 0.5%, basophils 0.1%, lymphocytes 13.7%, monocytes 83%), haemoglobin of 92 g/l, and platelets 10×10^9^/l. Lactate dehydrogenase (LDH) was increased to 2080 U/l (reference <245U/l). D-dimer was quantified at 1730 ug/L (reference range 0-700 ug/L). Ultrasonography revealed lymphadenopathy and splenomegaly. Bone marrow (BM) aspiration revealed 78% blast cells (**Figure 1A**). The blast cells were positive for cytoplasmic MPO, CD7, CD13, CD33, CD34, CD38 (dim), CD56 (partial), CD117, CD123, and HLA-DR, and negative for cytoplasmic CD3, cytoplasmic CD79a, CD1a, CD3, CD4, CD5, CD8, CD10, CD11b, CD14, CD15, CD16, CD19 and CD64 by flow cytometry (**Figure 1B**). Based on morphologic and immunophenotypical results, he was diagnosed with AML. Cytogenetic analyses revealed a normal male karyotype 46, XY.

**Figure 2:**
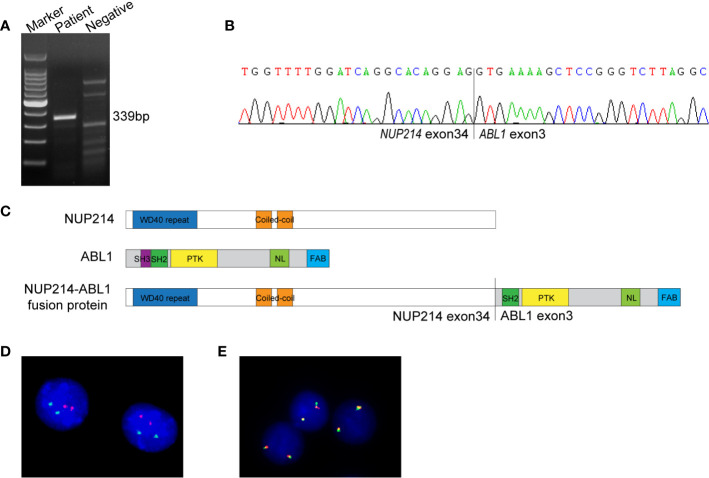
**(A)** RT-PCR for the *NUP214-ABL1* fusion transcript. The expected product size was 339 bp. **(B)** Sanger sequencing of the PCR product confirmed the *NUP214-ABL1* fusion gene of exon 34 of *NUP214* to exon 3 of *ABL1*. **(C)** The structure of *NUP214-ABL1* fusion protein. PTK, protein tyrosine kinase; NL, nuclear localization; FAB, F-actin binding. **(D)** FISH showed normal signal pattern using *BCR/ABL1* dual-color dual-fusion probe. **(E)** FISH showed two intact *ABL1* signals using an *ABL1* break-apart probe.

NGS (a panel of 88 genes) revealed that *CEBPA* (NM_004364.3) had double mutations (p.K304_Q305insL, and p.D75Gfs*33) and *NRAS* (NM_002524.5) had a point mutation (p.G13D). NGS also showed the presence of an *NUP214* (NM_005085.4)-*ABL1* (NM_007313.2) fusion gene (fusion of *NUP214* exon 34 and *ABL1* exon 3) (**Figure 1C**). Reverse transcription-polymerase chain reaction (RT-PCR) (primers: NUP214-E34-F GAGCAGCAGCAACACC, ABL1-E3-R TCACGCACCAAGAAGC) and sequencing of PCR products further confirmed *NUP214-ABL1* fusion (**Figures 2A, B**). The fusion protein of NUP214-ABL1 was shown in **Figure 2C**.

To further investigate the *NUP214-ABL1* fusion and explore whether there is amplification of the *ABL1* gene, interphase fluorescence *in situ* hybridization (FISH) was performed using the *BCR/ABL1* dual-colour dual-fusion probe and the *ABL1* break-apart probe (Zytovision, Germany). Both probes demonstrated normal signal patterns. No amplification or the split signal of *ABL1* was detected (**Figures 2D, E**).

With one cycle of conventional induction chemotherapy, which includes idarubicin and cytarabine, the patient achieved complete morphologic remission (CR) with a minimal residual disease (MRD) under 0.01%. After that, he was treated with high-dose cytarabine for consolidation therapy and is currently in his third phase of consolidation therapy. To date, he was in CR status.

## Discussion

In this study, we demonstrated the *NUP214-ABL1* fusion gene detected by NGS in a patient with AML. As far as we know, this is the first report of an AML patient with *NUP214-ABL1* fusion.

Identifying genetically targetable abnormalities is extremely important due to the proposal of targeted therapy in combination with chemotherapy and improved survival of patients. However, cryptic *ABL1* translocation is difficult to detect through conventional cytogenetic and FISH analysis, such as the *NUP214-ABL1* fusion in our case. The major cause might be that both *NUP214* and *ABL1* are located at the edges of 9q34, and FISH may not successfully detect the fusion of these two genes, as reported in a previous study (2). In this case, we revealed the presence of the *NUP214-ABL1* fusion through NGS and confirmed it by RT-PCR. Therefore, RT-PCR and some high-resolution sequencing, such as NGS, appear to be a very useful method to identify *NUP214-ABL1* fusions (10, 15). In addition, our case did not have extrachromosomal *ABL1* amplification, which is similar to the results of previous studies detected by FISH (2, 14). Notably, most *NUP214-ABL1* fusions were different exons of *NUP214* (from exons 23 to 34) fused to exon 2 of *ABL1* (1). However, the case described here involves *ABL1* exon 3, which is consistent with the other’s report (12).

Identification of molecular abnormalities by NGS could provide important prognostic and treatment information for AML patients, which has become a part of the clinical workup. Several molecular mutations, including *CEBPA, NPM1, FLT3, IDH1/IDH2, c-KIT, ASXL1, RUNX1, and TP53*, can refine prognostics groups, especially in patients with a normal karyotype (16). In this case, the patient had a normal karyotype, which is associated with intermediate risk of survival outcomes according to cytogenetic category in AML. He had *CEBPA* double mutations, *NRAS* point mutation, and *NUP214*-*ABL1* fusion gene, and he had no other adverse-risk genetic lesions. As we know, double *CEBPA* mutations are associated with favorable prognosis in patients with AML (17). Combining the results of cytogenetics and molecular mutations, the patient belongs to the group with favorable prognosis. Although the patient had the *NUP214-ABL1* fusion, the impact of this fusion on survival has not been determined. However, a promising test result was that the patient had no episomal amplification detected by FISH because previous studies reported that the presence of episomes implies a more radical disease process and a poorer prognosis in T-ALL (7, 8). Based on these clinical data, this patient may have a favorable or intermediate prognosis. Until today, the patient is still in remission with no signs of relapse.

The sensitivity of patients with *NUP214-ABL1* fusion to TKIs is controversial, as these subgroups of patients are rare (7–9, 14). Some reports have shown that TKIs might be effective for patients with *NUP214-ABL1* fusion (6, 10, 12, 13); however, they may be more suitable for use either in combination with other drugs (11–13) or as maintenance therapy after allo-HSCT (10). Some studies have demonstrated that *NUP214-ABL1*-positive patients show an initial favorable response to TKIs post relapse but can develop resistance to TKIs (8, 9). In this report, the patient received conventional AML-type chemotherapy, and he achieved a CR at the end of induction. Additionally, similar to our case, some patients with *NUP214-ABL1* fusion are treated by conventional chemotherapy without using TKIs and achieve a CR at the end of induction (9, 15). However, if this patient experiences relapse, we may consider adding TKIs, as previous studies suggested that *NUP214-ABL1-*positive patients could benefit from TKIs post relapse (12, 13). However, it remains unknown to what extent *NUP214-ABL1* is similar in ALL and AML and whether TKIs are also applicable to AML patients with the *NUP214-ABL1* fusion, as there is currently no report on this aspect. We look forward to more clinical experience to determine the sensitivity of *NUP214-ABL1-*positive AML to TKIs.

## Conclusion

To our knowledge, we describe the first case of *NUP214-ABL1* fusion gene in a patient with AML. This study emphasizes the need to detect *NUP214-ABL1* fusion gene in AML. The good result of this patient with conventional AML treatment regimen made it impossible to determine the sensitivity of *NUP214-ABL1* to TKIs. More case reports are needed to better study the sensitivity of NUP214-ABL1 fusion protein to TKIs in AML.

## Data Availability Statement

The raw sequence data reported in this paper have been deposited in the Genome Sequence Archive (Genomics, Proteomics & Bioinformatics 2017) in National Genomics Data Center (Nucleic Acids Res 2021), China National Center for Bioinformation/Beijing Institute of Genomics, Chinese Academy of Sciences. The accession number is: HRA000867. Please access it from the following link: https://bigd.big.ac.cn/gsa-human/browse/HRA000867.

## Ethics Statement

The studies involving human participants were reviewed and approved by the Research Ethics Committee of the First Affiliated Hospital, College of Medicine, Zhejiang University. The patients/participants provided their written informed consent to participate in this study.

## Author Contributions

H-PW wrote the manuscript. H-PW, J-JH, Q-YZ, LW, J-HL, and J-SH performed the research and analyzed the data. W-ZX provided samples and clinical data. JJ and H-HZ critically revised the manuscript. All authors contributed to the article and approved the submitted version.

## Conflict of Interest

The authors declare that the research was conducted in the absence of any commercial or financial relationships that could be construed as a potential conflict of interest.
